# Supplementary material for: Long-Term Survival Prediction Model for Elderly Community Members Using a Deep Learning Method
Source: Geriatrics (Basel). 2023 Oct 23;8(5):105. doi: 10.3390/geriatrics8050105 (PMC10606576; doi:10.3390/geriatrics8050105)
Supplement: Supplementary file 1 [file geriatrics-08-00105-s001.zip › geriatrics-2623693-supplementary.pdf]

Supplementary Table S1. Components of Deficit-Accumulation Frailty Index

Components	Definition	Data Source and Measurement	Scoring
**Medical history in the past year**			
Dysuria	Do you have dysuria?	Self-report	No (0)<br>Yes (1)
Fall	Have you fallen in the past 6 months?	Self-report	No (0)<br>Yes (1)
Gait disorder	Clinical observation of gait disorder	Clinical observation	No (0)<br>Yes (1)
Hearing impairment	Using pure-tone audiometry or whispered voice test (1) Pure-tone audiometry:	Objective measurement	No (0)<br>Yes (1)
Hypertension	'H35031', 'H35032', 'H35033', 'H35039', 'I10', 'I110', 'I119', 'I12', 'I130', 'I1310', 'I1311', 'I132', 'I15', 'I674', 'N262'	Claims Data	No (0)<br>Yes (1)
Stroke	'G45', 'G46', 'I60', 'I61', 'I62', 'I63', 'I64', 'I65', 'I66', 'I67', 'I68', 'I69'	Claims Data	No (0)<br>Yes (1)
Vision impairment	Visual acuity testing	Objective measurement	No trouble (0)<br>Blindness in one eye (0.5)<br>Blindness in both eyes (1)
**Biometric or laboratory measures**			
Alanine aminotransferase level, IU/L [a]		Laboratory test	≤35 (0)<br>36-45 (0.5)<br>≥46 (1)
Body mass index, kg/m^2 , or weight loss		Objective measurement	18.5 to 24.9 (0)<br>25.0 to 29.9 (0.5)<br>≥30 or ≤18.5 or weight loss 5% or more per

Components	Definition	Data Source and Measurement	Scoring
			year (1)
Bone mineral density (T-score) [b]		Diagnostic test	≥ -1.0 (0)<br>-2.4 to -1.1 (0.5)<br>≤ -2.5 (1)
Estimated GFR, ml/min/1.73m^2 [c]		Laboratory test	≥ 60 (0)<br>30 to 59 (0.5)<br>≤30 (1)
Estimated GFR, ml/min/1.73m^2		Laboratory test	≤ 99 (0)<br>100 to 125 (0.5)<br>≥ 126 (1)
Hemoglobin level, g/dL [d]		Laboratory test	Men:<br>≥ 13.0 (0)<br>12.0 to 12.9 (0.5)<br>≤ 11.9 (1)<br>Women:<br>≥ 12.0 (0)<br>11.0 to 11.9 (0.5)<br>≤ 10.9 (1)
Systolic blood pressure, mmHg		Objective measurement	≤ 119 (0)<br>120 to 139 (0.5)<br>≥ 140 (1)
Total cholesterol, mg/dL [e]		Laboratory test	≤ 199 (0)<br>200-239 (0.5)<br>≥ 240 (1)
**Physical health**			
Physical activity, MET-minutes/week		Self-report	≥ 1,017 (0)<br>558 to 1,016 (0.3)<br>1 to 557 (0.6)

Components	Definition	Data Source and Measurement	Scoring
3-m timed-up-and-go test, seconds		Objective test	0 (1)<br>≤ 10 (0)<br>11 to 19 (0.5)<br>≥ 20 (1)
**Psychological health**			
Have you dropped many of your activity or interests?		Self-report	No (0)<br>Yes (1)
Do you feel worthless the way you are now?		Self-report	No (0)<br>Yes (1)
Do you feel that your situation is hopeless?		Self-report	No (0)<br>Yes (1)
Do you think your memory is inferior to your friends or colleagues?		Self-report	No trouble (0)<br>Some (0.5)<br>A lot (1)
Do you think your memory has worsened over the last year?		Self-report	No trouble (0)<br>Some (0.5)<br>A lot (1)
Are there times when you feel memory is an impediment when doing important things?		Self-report	No trouble (0)<br>Some (0.5)<br>A lot (1)
Do others know that your memory has worsened?		Self-report	No trouble (0)<br>Some (0.5)<br>A lot (1)
Do you think that, when performing daily activities, you have become clumsier than before?		Self-report	No trouble (0)<br>Some (0.5)<br>A lot (1)
**Disability**			
Do you take a bath or shower without assistance?		Self-report	No (0)<br>Yes (1)
Do you get dressed without assistance?		Self-report	No (0)<br>Yes (1)

Components	Definition	Data Source and Measurement	Scoring
If a meal is prepared, do you eat without assistance?		Self-report	No (0)<br>Yes (1)
Do you get to the toilet without assistance?		Self-report	No (0)<br>Yes (1)
Do you prepare your own meals?		Self-report	No (0)<br>Yes (1)
Do you get to places out of walking distance, such as shops, neighbors, hospitals, and government offices, without assistance?		Self-report	No (0)<br>Yes (1)

Abbreviations: GFR, glomerular filtration unite; MET, metabolic equivalent for task.

[a] To convert to μkat/L, multiply by 0.0167.

[b] Bone mineral density was only measured for women.

[c] To convert to mmol/L, multiply by 0.0555.

[d] To convert to g/L, multiply by 10.0.

[e] To convert to mmol/L, multiply by 0.0259.
